# GanoCare® Improves Oil Palm Growth and Resistance against Ganoderma Basal Stem Rot Disease in Nursery and Field Trials

**DOI:** 10.1155/2020/3063710

**Published:** 2020-01-25

**Authors:** Nur Akmal Rebitanim, Mohamed Musa Hanafi, Abu Seman Idris, Siti Nor Akmar Abdullah, Hasmah Mohidin, Nur Zalikha Rebitanim

**Affiliations:** ^1^Laboratory of Plantation Crops, Institute of Tropical Agriculture, Universiti Putra Malaysia, 43400 Serdang, Selangor, Malaysia; ^2^Department of Land Management, Faculty of Agriculture, Universiti Putra Malaysia, 43400 Serdang, Selangor, Malaysia; ^3^Ganoderma and Disease Research of Oil Palm (GanoDROP) Unit, Biological Research Division, Malaysian Palm Oil Board (MPOB), 6, Persiaran Institusi, Bandar Baru Bangi, 43000 Kajang, Selangor, Malaysia; ^4^Faculty of Plantation and Agrotechnology, Universiti Teknologi MARA, 94300 Kota Samarahan, Sarawak, Malaysia; ^5^Faculty of Plantation and Agrotechnology, Universiti Teknologi MARA, 40450 Shah Alam, Selangor, Malaysia; ^6^Department of Chemical and Environmental Engineering, Faculty of Engineering, Universiti Putra Malaysia, 43400 Serdang, Selangor, Malaysia

## Abstract

Basal stem rot (BSR) caused by *Ganoderma boninense* is a major threat to sustainable oil palm production especially in Southeast Asia and has brought economic losses to the oil palm industry around the world. With no definitive cure at present, this study introduces a new fertilizer technology called GanoCare®, as an effort to suppress BSR incidence in oil palm. Experiments were carried out to evaluate the effect of GanoCare® on growth, physiology, and BSR disease suppression using sitting technique in the oil palm nursery stage. A follow-up using similar treatments was carried out in the field to test on severity of *Ganoderma* using baiting technique under natural condition. Treatments tested were 10 g/month and 30 g/three months given as pretreatment only or continuous treatment. Results showed that GanoCare® increased the height, bulb diameter, leaf area, chlorophyll content, photosynthesis rate, and fresh and dry weight of the leaf, bole, and root of oil palm seedlings in the nursery trial. Seedlings treated with GanoCare® exhibited reduced percentage of disease severity, incidence, and dead seedlings, compared to the control. In nursery and field, lowest percentage of dead seedlings due to *Ganoderma* was found in seedlings given combination of pretreatment and continuous treatment of 30 g/three months (T4) with 5.56 and 6.67%, while control seedlings significantly marked the maximum percentage of 94.45 and 93.33%. The most successful treatment in both nursery and field was T4 with disease reductions of 77.78 and 82.36%, respectively, proving that nutrients contained in GanoCare® are essential in allowing better development of a strong defense system in the seedlings.

## 1. Introduction

The current challenge of the oil palm industry is the occurrence of basal stem rot (BSR) disease caused by *Ganoderma boninense*, a soilborne fungus responsible for white rot in many tree species. It is the most severe oil palm disease, mainly in Malaysia and Indonesia, and its incidence has also been reported in southern Thailand, Papua New Guinea, Africa, Cameroon, Tanzania, Ghana, and Colombia [1, 2]. The BSR disease has markedly reduced the yearly harvest and caused palm oil loss up to USD 500 million per year [3]. Infected palms produced lower oil per bunch which explains large difference between the potential yield of 19 tons of crude palm oil/ha/year and the gained yield of 4 tons of crude palm oil/ha/year [4]. In Cameroon, BSR disease has caused about 53.2% of palm mortality in a first-generation planting [5], while in some Indonesian plantations, BSR disease has led to the death of palms up to 80% and reduced oil palm yield per unit area [6]. It has been estimated that 1% incidence causes USD 38 million loss annually to the Indonesian company [7]. In Malaysia, there is a reduction of 26 to 46% in fresh fruit bunch (FFB) yield at 31 to 67% of BSR disease [8]. Also, BSR incidence reported through survey was 3.71% and total area impacted was 59,148 hectares, with the most severe regions affected particularly in Perak, Johor, and Sabah [9].

Natural infection of *Ganoderma* in oil palm occurs as a result of root contact between healthy roots and diseased tissues in the soil [10], and the earliest symptom is usually observed from the fronds showing chlorosis, followed by necrosis. Such observations indicate that the root and bole system of oil palm has been infected, which resulted in decay of tissues and causes restriction of water and nutrient supply to the aerial parts of the plants. In young palms, external symptoms can be observed by the appearance of length reduction in the young unfolded leaves and mottling of lower fronds, sometimes with necrotic tips. As the disease progresses, spear leaves remain unopened and palms suffer from retardation in growth and take on pale appearance. Similar symptoms are observed in mature palms, with multiple unexpanded spear leaves and drooping of fronds forming skirt-like crown [11].

A variety of methods are being implemented by several oil palm plantations and smallholders in Malaysia as an integrated approach to control BSR disease. One of the practices involves soil mounding, a combination of cultural, organic, inorganic, and chemical treatments [12]. Surgery followed by soil mounding decreased the palm death from 34 to 2% in 24 months [13]. Breeding programs to develop oil palm progenies resistant to *Ganoderma* are also actively being undertaken by few research organizations for long-term solution. Application of chemical fungicides has constituted the major strategy for managing BSR disease, with soil drenching, trunk injection, or combinations of both being the standard methods used to apply fungicides in the field. Nonetheless, fungicides only assist in slowing the infection of *Ganoderma* and arising public alertness on the effect of fungicides due to the detrimental effects of nontarget organisms and environmental issues, such as ground water contamination [14, 15].

Owing to these limitations, developing another alternative which is cost-effective and environmentally safe by improvement of the oil palm defense system, through addition of required mineral nutrients, becomes an imperative solution and is seen as a promising approach to limit BSR. Nutrient supplementation to the soil is known to affect the susceptibility of plants towards some diseases caused by fungal pathogens [16]. In most cases, a balance of mineral nutrient added to the soil in the form of fertilizer improves the plants' ability to disease resistance [17]. Recently, Hasmah Mohidin (personal communication) reported that oil palm grown on peat soil under nursery conditions exhibited the best vegetative growth and BSR incidence reductions when given a combined primary macronutrient application of N, P_2_O_5_, and high K_2_O of 17.37, 17.37, and 41.34 g/plant. This corresponds to the highest activities of defense-related enzymes, namely, *β*-1,3-glucanase PAL, POX, and chitinase, found in the oil palm roots, thus supporting the role of macronutrients in inducing resistance towards *G. boninense*. Also, K affects plant metabolism and reduces the pathogen invasion by inducing the formation of thicker outer walls in epidermal cells [18]. In another study by Tengoua et al. [19], double combination treatments of micronutrients, namely, B + Mn and Cu + Mn, managed to alleviate BSR disease on oil palm planted under nursery condition with reductions of 16 and 24%, respectively. Supplementation of B with Mn may have retarded the growth of *G. boninense* in root cortex and bole tissues, while Mn in synergy with Cu may have improved the net uptake of Cu. A nursery study using beneficial nutrient by Najihah et al. [20] reported that oil palm seedlings supplemented with 1200 mg/L of SiO_2_ contributed the highest BSR reductions of 53%, with the lowest number of infected primary roots and bulb tissue lesions. Enhanced cellular features through Si deposits in the endodermal cells formed a mechanical barrier, thus limiting pathogen movement into the stems.

However, prior studies on this devastating disease have always been carried out based on nursery evaluation only and fundamental studies on the suppression of BSR disease in the field are very limited. The long-standing practices to carry out research studies have always been centered in the glasshouse or nursery rather than conducting field experiments, the real-life setting. It is critical to conduct trials in the field, as they are more expensive, labor-intensive, and time-consuming. Influence of the extraneous variables in the field is much larger than the nursery experiments due to the spatial variability in site factors, with uncontrollable climatic conditions which may greatly affect the growth and health of the plants.

Through nutrient supplementation, this study introduces a new fertilizer technology called GanoCare®. The GanoCare® is formulated to prevent BSR disease in oil palm. The fertilizer was derived by combining powdered empty fruit bunches (EFB) with “beneficial element.” Due to limitation of prior studies in the field, this paper provides a complete insight of combating *Ganoderma* in both nursery and field. Hence, the present study was undertaken in nursery trials to evaluate the effect of GanoCare® on vegetative growth, physiological parameters, and suppression of BSR disease of oil palm seedlings using sitting technique, and the scope was broadened by carrying out the GanoCare® treatments in the field stage based on seedling baiting technique.

## 2. Materials and Methods

### 2.1. Nursery Trials

Two experiments were conducted concurrently under a shaded nursery house in Field 2 (2°59′20.56″N, 101°42′44.42″E) of Universiti Putra Malaysia. In the first experiment, the effect of GanoCare® on vegetative growth and physiological study was evaluated. One-month-old oil palm seedlings (*Dura* × *Pisifera*) were collected from Federal Land Development Authority (FELDA) Agricultural Services Sdn. Bhd., Sungai Tekam, Pahang. At arrival, the seedlings were planted into small polybags (25 cm × 20 cm) and all seedlings except for control underwent pretreatment with GanoCare® for a period of three months (Table 1). The treatment rates applied were shortlisted from the preliminary optimization study that was conducted prior to this experiment. After three months of pretreatment, the seedlings were transplanted into bigger polybags (25 cm × 30 cm) containing 3 : 1 v/v topsoil : compost. The “continuous treatment” which represents the extended application of GanoCare® was carried out for eight months after transplanting.

In the second experiment, the effect of GanoCare® towards suppression of BSR disease was evaluated by challenging all seedlings with *G. boninense* (Isolate PER 71) after three months of pretreatment (Table 1) using sitting technique. The “continuous treatment” represents the extended application of GanoCare® after inoculation with *G. boninense* at a period of eight months. Both experiments were arranged in a randomized complete block design (RCBD) with six replicates for each treatment and three seedlings per replicate. Soil used throughout this study was taken from Puchong Field, Selangor, and classified as Serdang series, a loamy, siliceous, and isohyperthermic Typic Paleudult. Chemical properties of GanoCare® were pH of 6.15, organic C of 6.58%, and total N, P_2_O_5_, K_2_O_,_ Ca, Mg, Fe, Si, B, Cu, Zn, and Mn content of 0.68%, 1.56%, 0.99%, 4.32%, 0.48%, 1.38%, 12.0%, 18.85 ppm, 40.45 ppm, 96.20 ppm, and 298.4 ppm, respectively. Agronomic management practices, such as manuring with basic fertilizer (N-P_2_O_5_-K_2_O-MgO) [21], regular watering, and weeding, were carried out equally for all treatments.

#### 2.1.1. Assessment of Vegetative Growth and Physiological Parameters

At the end of experiment, growth parameters measured were (i) plant height, (ii) bulb diameter, (iii) leaf area, (iv) fresh weight of leaf, bole, and root, and (v) dry weight of leaf, bole, and root, while the physiological parameters collected were (i) chlorophyll content and (ii) photosynthesis rate. Before harvesting, plant height was taken by placing a measuring tape at the base of the plant to the tip of the first frond and bulb diameter was measured with a digital caliper. The amount of chlorophyll was measured on the third leaf with a chlorophyll meter (Minolta SPAD-502), and the net photosynthesis rate of the third leaves were measured between 8:00 and 11:00 h using an open gas exchange system of photosynthesis analyzer (Li-6400 Lincoln, Nebraska, USA). At harvest, seedlings were uprooted and the leaf, bole, and root were separated for fresh weight measurement. Then, they were washed with distilled water and weighed for fresh biomass. Leaf area was then measured using the leaf area meter (LI-3100C Area Meter). Finally, the leaf, bole, and root were oven-dried at 70°C for one week, until their weight became constant and the dry weight was taken.

#### 2.1.2. Preparation of *G. boninense* Inoculum and Inoculation of Seedlings with *G. boninense*

Preparation of *G. boninense* rubber wood block (RWB) was done according to the general method used by the GanoDROP Unit of Malaysian Palm Oil Board (MPOB) [22]. Freshly cut RWBs, each with the size of 6 cm × 6 cm × 6 cm, were oven-dried at 80°C for 48 hours and autoclaved for 30 minutes at 121°C and 103.4 kPa psi. This sterilization step was repeated twice. Each RWB was placed in double-layer polypropylene plastic bags to which 65 mL of malt extract agar (MEA) was poured onto the block. The plastic bags were then autoclaved for 30 minutes at 121°C and let cooled. During the cooling process, the RWB in the polypropylene bag was rotated to ensure that it was fully covered with MEA before leaving overnight to let the MEA completely solidify. The RWBs were inoculated with 7- to 10-day-old *G. boninense* subcultures grown on potato dextrose agar (PDA) at one plate per two blocks. The pure culture of *G. boninense* (Isolate PER 71) was previously supplied by the MPOB. The plastic bags of inoculated RWBs were then secured with a rubber band to prevent contamination and incubated in the dark at 28°C ± 2 for three months to allow colonization of *G. boninense* mycelium.

For the artificial infection, a sitting technique was used to inoculate oil palm seedlings with *Ganoderma* RWB inoculums. The root of the seedlings was firmly placed in contact with the RWB inoculums standing on one third of a soil mixture (3 : 1 v/v topsoil:compost) in a polybag (25 cm × 30 cm). Once in place, the seedling was covered with the remaining soil. For T3 and T4 treatments, the application of GanoCare® was carried out after the inoculation.

#### 2.1.3. Assessment of Pathological Parameters

**Figure 1:**
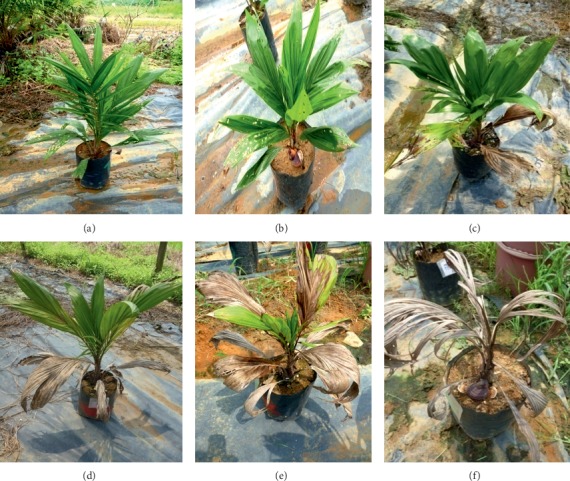
Disease classes to measure DSIF of oil palm seedlings: (a) class 0; (b) class 1; (c) class 2; (d) class 3; (e, f) class 4.

For external symptoms, disease severity of foliar index (DSIF) was recorded at eight-week intervals after inoculation until the 8^th^ month of inoculation. The disease classes (Figure 1) were described according to Abdullah et al. [23] and Ilias [24] with slight modifications: 0 = healthy palm, 1 = palm with presence of white fungal mass on any part of plants without necrotic or chlorotic leaves, 2 = chlorotic and/or necrotic leaves (<25%), with or without the presence of basidiomata on any part of plant, 3 = chlorotic and/or necrotic leaves (<75%), with or without the presence of basidiomata on any part of plant, and 4 = chlorotic and/or necrotic leaves (>75%), with or without the presence of basidiomata on any part of the plant or dead seedling. The DSIF was expressed according to the disease severity (DS) formula given by Liu et al. [25].

(1) $$\begin{array}{c} \text{Disease Severity}\left(\%\right)=\frac{\sum \left(\text{Number of seedings in the scale}\times \text{Severity scale}\right)}{\text{Total number of seedlings assessed}\times Η\text{ighest scale}}\times 100. \end{array}$$

**Figure 2:**
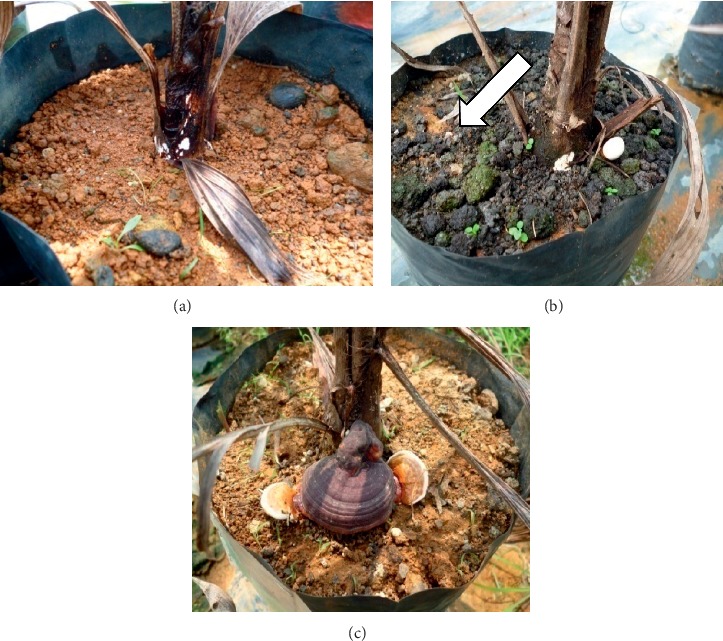
External infection usually started with the appearance of (a) white mycelium, which, as the disease infection progressed, developed into (b) white button (arrow) and finally (c) full fruiting body or basidiomata.

Eight months after inoculation, disease incidence (DI) was assessed based on the number of visibly diseased seedlings (emergence of foliar symptom, white mycelia, white button, or fruiting body on the oil palm seedlings (Figure 2) relative to the total number of seedlings. With the DI data, disease development was further expressed based on the AUDPC, which signifies the amount of disease developed over time for each treatment. The AUDPC was measured according to Campbell and Madden [26] as follows:

(2) $$\begin{array}{c} \text{AUDPC}=\overset{n-1}{\underset{1}{\sum }}\left(\frac{Yi+Yi+1}{2}\right)\left(ti+1-ti\right), \end{array}$$

where *n* is the number of assessment times, *Y* is the disease incidence, and *t* is the time of observation. The slopes of curves were attained by transforming DI data with the monomolecular model (Monit) by Campbell and Madden [26]. Disease reduction (DR) was deduced from the AUDPC by the following formula:

(3) $$\begin{array}{c} \%\text{DR}=\left[\frac{\left(\text{AUDPC control}-\text{AUDPC treatment}\right)}{\text{AUDPC control}}\right]\;\times \;100. \end{array}$$

To confirm the external symptoms, surviving seedlings were assessed for *Ganoderma* internal symptoms at the end of experiment. The seedlings were uprooted, longitudinally sectioned to visually assess internal symptoms according to the bole and root tissue decay. Percentage of bole or root decay was calculated as follows:

(4) $$\begin{array}{c} \%\text{ tissue decay }=\;\left(\frac{\text{infected tissue area}}{\text{total tissue area}}\right)\times 100. \end{array}$$

The decay of bole and root tissues damage was then classed based on the adapted scale from Nur Sabrina et al. [27] as follows: 0 = healthy, no rotting of tissues, 1 = less than 25% rotting of tissues, 2 = 25 to 50% rotting of tissues, 3 = more than 50% rotting of tissues, and 4 = more than 80% rotting of tissues or total rotting. Based on these scores, the disease severity of bole and root index (DSIB and DSIR) was expressed according to the disease severity formula described previously for external symptoms [25].

### 2.2. Bait Seedling Trial in the Field

Experiment was conducted in an oil palm plantation field in Peringkat 13 (4°14′20.80″N, 100°84′19.30″E) in Seberang Perak, Malaysia. The field belongs to the Federal Land Consolidation and Rehabilitation Authority (FELCRA) Plantation Services Sdn. Bhd. and consisted of mature oil palms that were planted in 1988. The soil in the field was mainly composed of local alluvium of Telemong Akob series, and it is classified as Entisols or coarse, loamy, mixed, isohyperthermic Typic Dystrudepts (USDA Soil Taxonomy, 2014). The field was severely infected with BSR and thus has met our criteria to be selected as our study site.

The experimental design used in this experiment was a completely randomized design (CRD) with five treatments (Table 1). Each treatment was replicated five times with three seedlings per replication. Pretreatment was carried out in Field 2, Universiti Putra Malaysia, in which eight-month-old oil palm seedlings (*Dura* × *Pisifera*) were pretreated with GanoCare® during a six-month duration. After six months of pretreatment, all the seedlings were transferred and planted in the plantation field in Seberang Perak, where some of the seedlings received continuous treatment. “Continuous” treatment indicates the extended application of GanoCare® after the pretreated seedlings had been planted next to diseased palms in the field. This field trial was conducted for a duration of 21 months.

Baiting of seedlings started with the selection of *Ganoderma*-infected oil palm trees. Selection was carried out based on their external visual conditions, such as occurrence of multiple spear leaves and pale and collapse leaf canopy and appearance of fruiting body at the base of the stem. Severely infested oil palm that exhibited three to four or more fruiting bodies at different regions of the palm base was chosen as experimental palm [28]. Then, the GSM test was performed by plating fragments of fruiting bodies from the tree base on the GSM, and development of brown medium after five days confirmed the *Ganoderma* infections [29]. After selection of infected trees was done, seedlings were planted next to the tree, with a seedling per tree. Each planting hole with depths of about 0.3–0.5 m was dug adjacent (0.3–0.5 m) to the region where palm tree base was severely infested by *G. boninense.* The infected tree functioned as a natural inoculum, while the seedling placed next to it served as bait for *G. boninense*. After the seedlings were planted, supplementation of GanoCare® was continued for T3 and T4 treatments. Other agronomic managements, such as basic fertilizer application, and maintenance of field, such as weeding, were according to normal recommended field practices.

#### 2.2.1. Assessment of Pathological Parameters in Field Trial

External and internal disease assessments were carried out similarly as described in Section 2.1.3. External symptoms were recorded at nine months after planting in the field when the visible symptoms started to appear. They were assessed at three-month intervals up to 21 months after planting in the field. At the end of experiment, the surviving seedlings were assessed for the BSR internal symptoms.

### 2.3. Statistical Analysis

Data for all three experiments from both nursery and field were analyzed separately by using ANOVA procedure using SAS 9.2 software at *p* < 0.05, and means were tested using least significant difference (LSD) if the ANOVA was noted to be significant. Percentage data were arcsine transformed before analysis.

## 3. Results and Discussion

### 3.1. Effect of GanoCare® on Growth and Physiological Parameters of Oil Palm

Growth parameters such as height, bulb diameter, leaf area, bole fresh weight, and dry weight of the leaf and bole (Table 2) were significantly enhanced when oil palm was treated with GanoCare® treatments. Despite the insignificant differences in treatments of fresh weight of leaf and root and also dry weight of root, control (CT) seedlings recorded the minimum values for these parameters indicating that application of GanoCare® proved to provide a positive effect in promoting oil palm growth. Maximum palm height, bulb diameter, and leaf area data were obtained from T4 seedlings, with 23%, 11%, and 29% significant increment relative to untreated seedlings. Application of GanoCare® was shown to increase the dry weight of leaf, bole, and root, with more pronounced increment recorded in T4 seedlings with 22%, 49%, and 36% relative to control seedlings.

**Figure 3:**
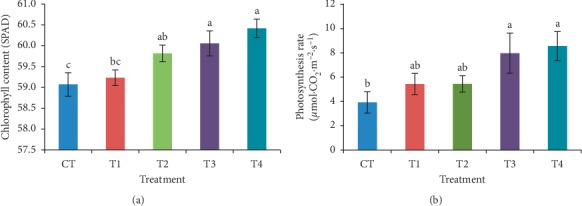
Effect of GanoCare® on (a) chlorophyll content and (b) photosynthesis rate of oil palm seedlings in nursery trial. Values are means of 6 replicates with vertical bars representing standard errors, and the same letters are not significantly different according to least significant difference (LSD) at *p* < 0.05.

The potential of GanoCare® as a oil palm growth enhancer was further confirmed with the results of physiological parameters. Application of GanoCare® not only enhances the plant vigour, but the leaves of treated seedlings were observed to be thicker, darker green, and more erect than the leaves of the control seedlings. The strong stems and erect leaves of treated seedlings had captured more sunlight and contributed to the enhancement of crop photosynthesis, hence showing the significant increment in photosynthesis activity and chlorophyll content (Figure 3) of the treated seedlings compared to the control seedlings. Three highest chlorophyll content values came from T4, T3, and T2 in the descending order, with maximum chlorophyll content (60.42 SPAD) observed for T4, which was 2.3% significantly higher than that of the control seedlings. The same trend was recorded in the data of photosynthesis rate, revealing that the maximum photosynthesis rate of 8.56 *µ*mol·CO_2_·m^−2^·s^−1^ was achieved when T4 was applied, followed by T3, T2, and T1, with insignificant differences between them. The minimum photosynthesis rate was recorded by control seedlings, which was statistically at par with T1 and T2.

The improved growth and physiological performance of the oil palm seedlings were attributed to the mineral nutrients, which are the N, P, K, Ca, Mg, Fe, Si, B, Cu, Zn, and Mn that are contained in the GanoCare® product. For instance, N gives an intense effect on rapid development and growth of plants under most agro-ecosystems [30] and it is responsible in leaf formation, size enlargement, and yield of the plants [31]. Application of N and P at the main nursery improved the production of leaves and stem girth of oil palm [32] and have also been reported to increase the height, stem girth, frond number, chlorophyll content, and leaf area of the 9^th^ frond of 1-year-old oil palm [33,34]. High content of micronutrient Mn in GanoCare® might also have assisted the palm's growth as its role in cell division and extension, especially in roots [35], and is essential for splitting water during photosynthesis. Presence of K and Mg in GanoCare® may have enhanced the photosynthesis rate and chlorophyll content of oil palms. In a study, increased application levels of K improved the light interception, hence enhancing the dry matter and yield of oil palm by 38% [36]. Mg, which is the central element of chlorophyll, aids in efficient photosynthesis, and a study has proved that application of Mg as a kieserite to oil palm increased the oil-to- bunch ratio by 1 and 1.8% [37].

### 3.2. Effect of GanoCare® on Pathological Parameters

#### 3.2.1. Disease Severity and Percentage of Infected Tissues of Oil Palm Seedlings

**Figure 4:**
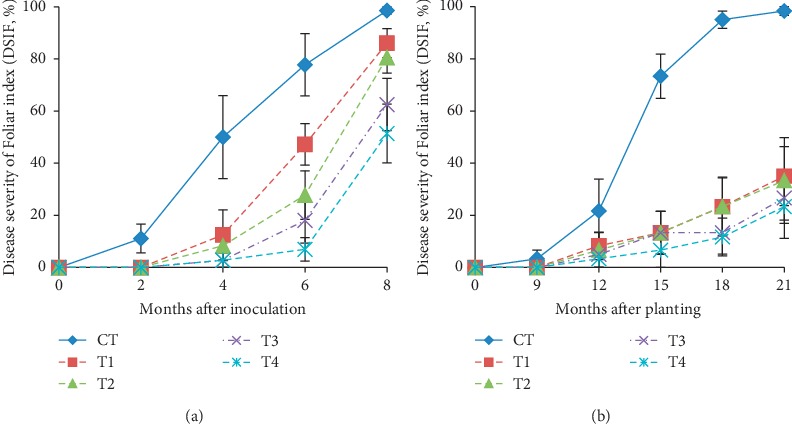
Disease severity of foliar index of oil palm seedlings in (a) nursery and (b) field trial. Values are means of 6 replicates in nursery and 5 replicates in field trial with vertical bars representing standard errors.

Our pathological study was first carried out in the nursery using artificial inoculation, with a follow-up in the field trial using baiting technique which signifies natural infection of *G. boninense*. Early external symptoms of BSR infection was prominently observed from the change in foliar appearance. The first foliar symptoms were observed in control seedlings as early as two months after seedling was artificially inoculated in the nursery, while it took nine months for the foliar symptoms to make its first appearance in the field trial (Figure 4). This study observes that foliar symptoms were exhibited by progressive yellowing and desiccation of fronds, from older to younger leaves. The yellowing and drying of fronds usually initiated from the frond tips, after which the entire innermost whorl of frond progressively dried out and led to necrosis. Desiccation of fronds was attributed to the impact of *Ganoderma* that induces degradation of lignin and cellulose of the stems, consequently restricting water uptake to the upper plant parts [38].

Nursery results revealed that at two months after inoculation, seedlings that were given GanoCare® treatment (T1, T2, T3, and T4) did not yet develop any signs of infections and were indexed as 0%, while control seedlings were already affected by *G. boninense*, with disease severity of foliar index (DSIF) as high as 11.11%. At six and eight months after inoculation, the maximum DSIF was observed in control seedlings (77.78% and 98.61%), while the minimum DSIF was recorded for T4 (6.94% and 51.39%). Similar trend was observed in the results from the field trial. At 18 and 21 months after planting in the field, control seedlings exhibited the highest DSIF of 95.0% and 98.33%, which were significantly different from other treatments, indicating that good BSR suppression can be achieved through supplementation of GanoCare®.

**Figure 5:**
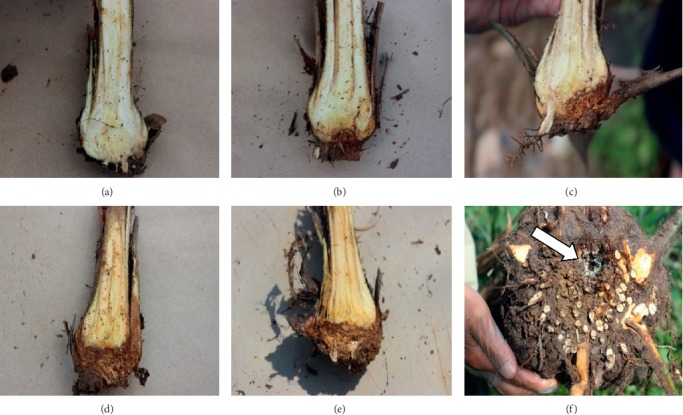
Degree of bole infection of oil palm seedlings: (a) healthy bole; (b) less than 25% decay of bole tissues; (c) tissues decayed within 25% to 50%; (d) more than 50% of tissues decayed; (e) 100% or total rotting of bole tissues; (f) a region of bole rotted and became hollow (arrow) with appearance of white mycelia mass.

Infection of *G. boninense* begins with the attachment of hyphae to the root surface. Following the penetration of fungus into the root tissues, the fungus will grow along the roots and the infection will eventually outstretch till it reaches the bole. Thus, destructive sampling was carried out at the end of both trials to assess the extent of internal damage in the roots and bole regions. Our findings show that different treatments given to the oil palm impacted different levels of host resistance towards BSR infection, hence the difference in proportion of root and bole decay. In the nursery and field trials, the highest level of root rot was observed in the control seedlings and this corresponds to the highest disease severity of root index (DSIR) (Table 3). When cross-sectioned, the infected roots were easily distinguishable from the healthy roots by its rot and dry appearance with light to dark brown discoloration, whereas healthy roots exhibited ivory to cream-colored tissues. When uprooted, some infected roots possess thin white mycelia masses (Figure 5(f)) on their root surface, typically below the bole region. Longitudinal sectioning of infected bole shows the brown discoloration of tissues implying the progressive decay of *Ganoderma* from the roots to the boles. Different proportions of bole tissue decay gave different degrees of infection (Figure 5). Our study observed that tissues of infected boles exhibited shrinkage and lack of moisture compared to the healthy boles which indicated that uptake of water is restricted due to rotting of xylem. Infected palms were subjected to water stress due to *Ganoderma* which resulted in injury to the root and vascular transport system [39]. Based on the nursery results, control seedlings showed the maximum percentage of root (99.65%) and bole (99.44%) decay which corresponds to the DSIR and disease severity of bole index (DSIB) values with 100% of root and bole infections by *G. boninense*. The lowest DSIR (59.72%) and DSIB (37.5%) were observed on T4 seedlings. Similar trend of results was observed in the field trial with highest DSIR and DSIB exhibited by the control seedlings, with both displaying the same value of 95.0%, which was significantly different with T1, T2, T3, and T4.

#### 3.2.2. Disease Incidence

**Figure 6:**
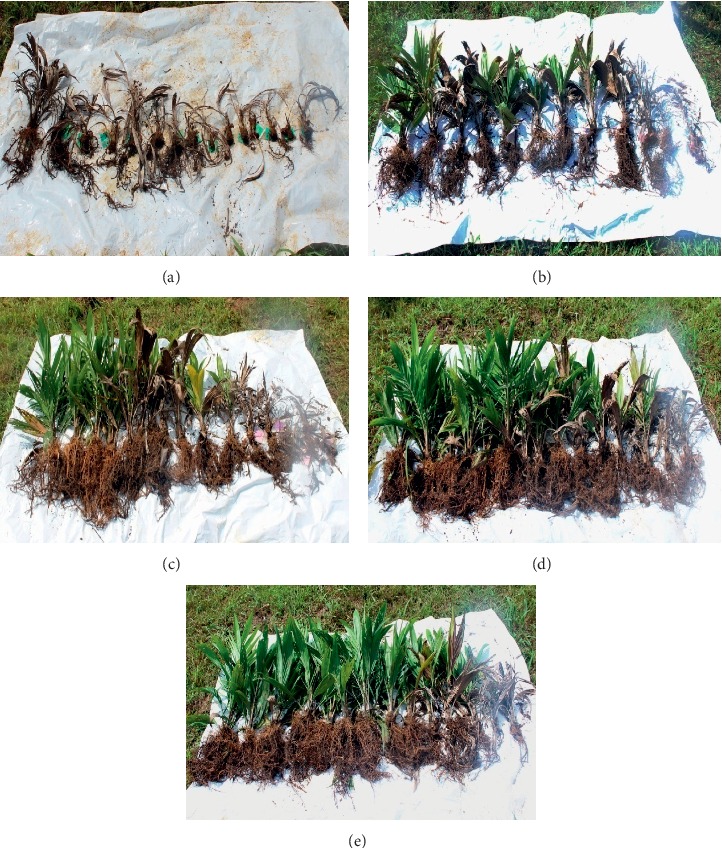
Effect of GanoCare® on disease development of (a) control, (b) T1, (c) T2, (d) T3, and (e) T4 seedlings over duration of 8 months in nursery.

**Figure 7:**
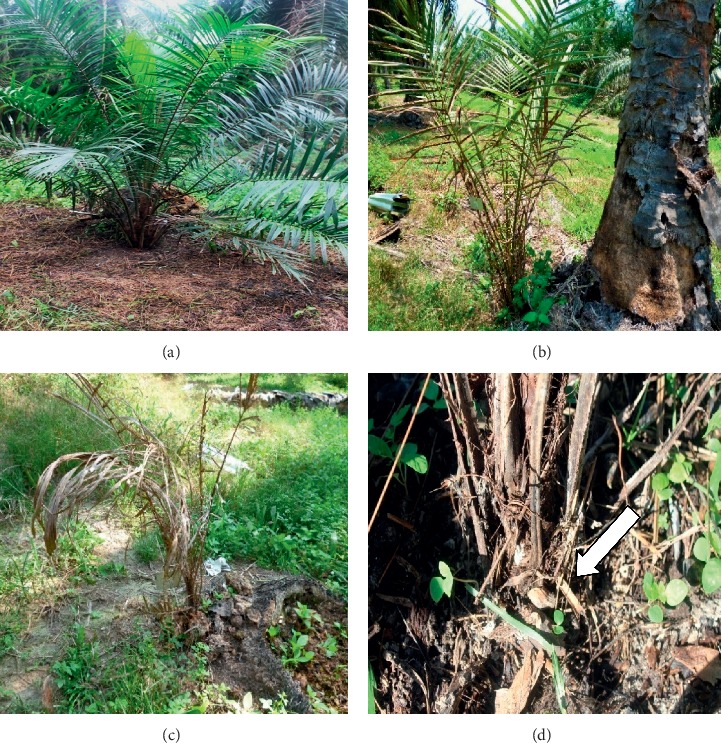
Bait seedlings in the field: (a) GanoCare®-treated seedling with no visible symptoms of BSR disease at 21 months after planting; (b) more than 75% of chlorotic leaves due to *Ganoderma* infection; (c) dead seedling due to Ganoderma infection; (d) growth of fruiting body (arrow) at the base of the seedling.

Evaluation of disease incidence (DI) was recorded according to the presence of foliar symptoms and development of white mycelia and basidiomata. In the nursery trial, first emergence of mycelia was observed in control seedlings as early as two months after inoculation. The early emergence of mycelia occurred concurrently with the foliar symptoms and as the infection progressed, the mycelia developed into fully developed basidiomata. Control seedlings were the most severely infected with 100% of DI (Table 4; Figure 6) recorded after eight months of inoculation in the nursery trial. The DI for T1 and T2 seedlings was observed to be statistically at par with each other with the value of 94.4%, which was also statistically equal to the DI of T3 and control seedlings. Meanwhile, T4 exhibited the lowest DI (72.2%), even though the result was statistically at par with all other treatments except control. Unlike the nursery trial, very limited occurrence of white mycelia and basidiomata was observed from the seedlings planted in the field. Hence, the DI for nearly all of the seedlings in the field was assessed based on their foliar symptoms only (Figure 7). At 21 months after planting, T1, T2, T3, and T4 statistically had similar effects on the DI, with the lowest DI (26.67%) observed for seedlings subjected to continuous treatment at 30 g/three months (T4), followed by T3 (33.33%), while the seedlings subjected to pretreatment alone (T1 and T2) recorded the same DI (40%), respectively.

#### 3.2.3. Dead Seedling

**Figure 8:**
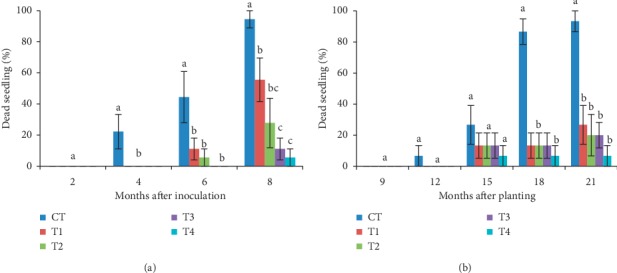
Percentage of dead seedlings in (a) nursery and (b) field trial. Values are means of 6 replicates in nursery and 5 replicates in field, with vertical bars representing standard errors, and the same letters are not significantly different according to least significant difference (LSD) at *p* < 0.05.

Four months after inoculation in the nursery trial, all GanoCare®-treated seedlings (T1, T2, T3, and T4) were alive, while control seedlings significantly exhibited 22.22% death incidents (Figure 8). Eight months after inoculation, T4 exhibited the lowest (5.56%) dead seedling, followed by T3 (11.11%), with insignificant differences between them, while control seedlings recorded the maximum (94.45%) percentage of dead seedlings. For the field trial, dead seedlings were observed starting at twelve months after planting. Although there were no significant differences in the dead seedling data at 12 months after planting, all seedlings that were given GanoCare® treatments were alive, while control seedlings exhibited 6.67% of death incidents. At 18 months after planting, death incidents of control seedlings rose up to 86.67%, which was significantly different with other treatments. Similar trend of data was observed at 21 months after planting, with the maximum (93.33%) dead seedling recorded by the control which was significantly different from all other treatments, while minimum (6.67%) dead seedling was recorded by T4. Percentage of dead seedling for T4 was observed persistent from 15 months after planting till the end of experiment.

#### 3.2.4. Area under the Disease Progress Curve and Disease Reduction

The AUDPC is the amount of disease developed in each treatment over time. High disease symptom progress is indicated by high AUDPC. Meanwhile, lower AUDPC value indicates the effectiveness of that treatment in suppressing the disease [26]. In the nursery study, T4 seedlings exhibited significantly lowest (105.56 unit^2^) AUDPC, followed by T3 (161.11 unit^2^), while the control seedlings had significantly the highest AUDPC of 450 unit^2^ (Table 4). Similar trend of results was observed in the field trial, with T4 seedlings exhibiting the lowest AUDPC value, while T1 and T2 recorded the similar value which is 220 unit^2^. The control seedlings significantly exhibited the highest AUDPC value of 800 unit^2^. This strongly suggests that seedlings which were untreated with GanoCare® developed a higher amount of BSR disease incidence than the treated seedlings. These AUDPCs correspond with the DR data, in which the highest DR for both nursery and field were indicated by T4 seedlings with 77.78% and 82.36% reduction compared to the control, followed by T3 with 63.47% and 78.93% reduction.

The nutrient supplementation from GanoCare® has caused the seedlings to acquire a level of tolerance towards the physical destruction by *G. boninense*. In plant defense mechanism, nutrients play a role as integral component of activators, regulators, and inhibitors of metabolism, electron carriers, and also part of cells, substrates, and enzymes [40]. Macronutrients and micronutrients have been recognized to be involved in plant development and their yield, hence showing the level of resistance towards disease. Nutrient application may affect the disease level by affecting the (i) microclimate of the plant, thus influencing the infection and sporulation of the pathogen, (ii) tissues, cell walls, and biochemical composition of the plants, (iii) growth rate of the host, thus allowing seedlings to escape infections in their most susceptible stage, and (iv) soil environment that may influence the pathogen [41]. Application of nutrients may either increase or decrease disease development by affecting the anatomy, morphology, growth patterns, and chemical composition of the plants [42]. In most situations, a balanced nutrient in both soil and plant enhances the plant's ability to disease resistance [17].

The presence of essential macronutrients in GanoCare® may contribute to suppression of BSR disease of the seedlings. In a trial on the marine alluvium of the Briah-Selangor association, application of N fertilizer reduced the BSR incidence in oil palm [8]. However, in another study by Tayeb and Hamdan [43], the BSR incidence increased as the N fertilizer increased. Increasing N uptake may either increase or decrease the disease incidence, depending on the crop species and the source of N used. Phosphorus is an essential element for plant growth. Adequate fertilization of P may increase physiological resistance and reduce the intensity of several infectious diseases in crops [44]. In the Paya Lang trial, on the lateritic Malacca series soil, fertilization of oil palms with P significantly reduced the BSR incidence [43]. The root growth assisted by P fertilization may have allowed the crop to escape attack by soilborne pathogens [45]. In addition, fertilization with K reduced the incidence of bacterial blight in rice, leaf rust in wheat, and pod rot in soybean. Enhanced plant resistance to disease was attributed to the effect of K in increasing the thickness of the epidermal cell wall, thus preventing disease attacks [45].

The abundant presence of Ca and Si in the GanoCare® may have given the greatest contribution to the enhanced resistance towards the BSR disease. Research by Sariah et al. [46] reported that the foliar symptoms, number of fruiting bodies, and lesioned roots and bole decreased when seedlings were grown in soils supplemented with 7.5 g CaNO_3_ at one month before challenged with *G. boninense*. The Ca-supplemented seedlings contained well-developed lamellae due to accumulation of Ca pectate, which assists in stabilizing the cell walls and enduring degradation by the pathogen enzymes. The positive effect of Ca supplementation in reducing the incidence of BSR in oil palm has been further demonstrated by Nur Sabrina et al. [27]. At six months after inoculation with *G. boninense*, combination of Ca and Cu gave the best disease suppression compared to other treatments. The supplementation of Ca assisted in the lignification process, which increased production of two lignin-related enzymes, peroxidase (POD) and laccase, thus resulting in amplified lignin production. Lignification protects the cellulose fibers from biological degradation and chemical injuries [47], where lignin acts as the barrier that will slow down the penetration of the pathogen during fungal attack. Beneficial effects of Ca which involves improving the soil structure and stabilizing the cell membranes also help to lower the probability of *Ganoderma* attachment [27]. For Mg element, this nutrient has been reported to enhance the tolerance of tissue towards maceration by pectolytic enzymes produced by soft rot bacteria [48], but apart from this, the effects of Mg on pathogenesis have not been well studied.

The beneficial effect of Si fertilizer application has been reported to suppress many diseases on various kinds of crops including rice, sugarcane, banana, barley, muskmelon, and corn [49]. Accumulation and polymerization of Si in the form of silicic acid in the epidermal cells acts as a mechanical barrier against fungal penetration [50]. Deposition of Si in the cell walls can be associated with lignin-carbohydrate complexes [51]. Silicon also mediates some physiological changes to improve the resistance towards disease infestation [50]. Induced resistance to plants by Si is a result of accumulation of antifungal compounds, such as phytoalexins and pathogenesis-related proteins in plants [52]. Inoculation of *M. grisea* on two isogenic susceptible and resistant lines of Si-treated rice resulted in reduced disease severity and increased activities of POD, PPO, and PAL in both lines [53].

The effect of several combinations of micronutrients towards BSR incidence in oil palm has been demonstrated by Tengoua et al. [19]. The double combinations of B + Cu, B + Mn, and Cu + Mn have shown suppression of BSR disease. Their findings have suggested that supplementation of single Mn increased BSR infections was possibly due to reduced beneficial effect of Mn through Mn oxidation by *Ganoderma* Mn-oxidizing enzymes. In different studies, fertilization of Mn has been reported to reduce the infection of various diseases including wilt in cotton, scab in potato, blast in rice, and leaf spot in sugar beet [54]. Under Mn-sufficient conditions, Mn reduces infection of the take-all disease of cereals caused by *Gaeumannomyces graminis* by increasing deposition of lignin [55]. Such effects were attributed to the role of Mn in controlling the lignin and suberin biosynthesis, through activation of several enzymes of the shikimic acid and phenylpropanoid pathways [42]. These factors play important barriers towards fungal pathogen invasion.

Boron in the GanoCare® might also be a contributor to the enhanced resistance towards *Ganoderma*, which contributes to cell wall development and nucleotide synthesis and assimilates translocation [56]. The role played by B includes the formation of carbohydrate-borate complexes which control carbohydrate transport and cell wall protein metabolism. When a crop is deficient in B, cell walls tend to swell and split, and the intercellular space is weakened. As a result, physical barrier is vulnerable to infection and expansion of the disease. Fertilization with B has been reported to reduce the risk of *Plasmodiophora brassicae* in *Brassica* species, and this is associated with the role of B in lignification and phenol metabolism [57]. The enhanced resistance is due to B's roles in sustaining the rigidity of cell wall structure and integrity of plasma membrane [18].

Another essential micronutrient in GanoCare® is Zn. Application of Zn to crops has exhibited suppressive effects on soilborne, bacterial, and viral diseases [58], including crown and root rot in wheat, smut in corn, leaf spot in alfalfa, and powdery scab in potato. Zinc plays a role in affecting growth and secondary metabolism of fungus and indirectly influenced the host susceptibility [59]. Similarly, fertilization with Cu has been reported to reduce the risk of various diseases including leaf spot and root rot in pea, blast and sheath rot in rice, rust in sugarcane, and wilt in cotton [41]. Copper acts as a regulator in many enzyme systems involved in plant defense and participates in the production of antimicrobial compounds. Adequate amount of Cu in plant and soil systems may act as a bactericide and fungicide in controlling disease pathogens. Fe plays a role in production of carbohydrate, chlorophyll synthesis, and cell respiration, and Fe deficiency in plants is characterized by interveinal chlorosis in younger leaves [31]. In Fe-deficient soil, foliar fertilization with Fe reduces the risk of various diseases including smut in wheat, *Spaeropsis inalorum* in pears and apples, and *Fusarium* patch disease in turf grass. In addition, application of Fe was able to correct virus symptoms in camellia and enhanced resistance of cabbage to *Olpidium brassicae* [60]. Disease suppression may be due to the presence of microbial siderophores that increase the availability of Fe in the soil, which then indirectly limit the growth of pathogens [61].

The powdered EFB used in the production of GanoCare® plays a role as binding agent or as glue to hold the beneficial elements together. The usage of EFB is an effort to adopt more environmental-friendly approaches in providing nutrients to the palms. Besides sustaining soil fertility, the abundancy of EFB as main solid waste from palm oil extraction process has made EFB, a renewable low-cost material, thus a preferred source in this study. It is vital to manage this organic waste properly through recycling, and in this study, we have converted it into a valuable market product, for the use of plantations in suppressing the risk of *Ganoderma*.

The present results showed that the lowest percentage of BSR incidence in the field was found in T4 treatment. While the result is positively encouraging as compared to control, the application of T4 treatment in real plantation will imply that the oil palm planters will need to face the risk of losing palms of about 25% of the population due to the *Ganoderma*, hence bringing economic loss to them. However, the present study was performed under severe confirmed *Ganoderma* infection at the planting hole. Planters may reduce this risk by carrying out longer pretreatment on younger seedlings, considering that the seedlings used in the current study were subjected to six-month pretreatment that may not be sufficient to provide maximum protection from *Ganoderma* disease. Increasing the pretreatment period might develop seedling with a stronger defense system to accomplish successful field establishment. Additionally, treatment of GanoCare® as part of the integrated management in controlling *Ganoderma* disease could be a viable addition to the foundation of good land preparation. As reported by Idris et al. [62], sanitation or removal of infected palm could significantly reduce the *Ganoderma* infections to 0% compared to control (without sanitation) at 87.5% after two years of planting in *Ganoderma*-infected areas. It was well noted by several previous studies that integrated disease management (IDM), which combines biological, cultural, physical, and chemical control strategies in holistic approaches compared to using a single strategy proved to be more effective, sustainable, and gaining momentum.

Vigorous seedlings with an enhanced defense system at transplant will prevent high mortality rate in the field, not only due to BSR disease alone but also due to possible infestations by pests such as rats and beetles. Hence, good establishment during the stage is vital for the planters to have the potential high fruit yields for the next 20 years.

From our findings, better enhancement of growth, physiological parameters, and reductions in BSR disease was found in seedlings that were given a combination of pretreatment and continuous treatment (T3 and T4) compared to seedlings that were given pretreatment alone (T1 and T2). Overall, the combination of pretreatment and continuous treatment with 30 g of GanoCare® at three-month intervals (T4) was the most effective treatment in promoting oil palm growth and reducing the BSR disease development with reductions of 77.78% and 82.36% in nursery and field trial. In developing a good fertilizer programme, the frequency of application is essential to be determined. This study suggests that pretreatment of GanoCare® without continuous application was not sufficient enough to allow the development of a strong defense system in the plant, hence showing the low potential to reduce *Ganoderma* incidence. In addition, planters are highly recommended to use T4 treatment for their plantations. Not only T4 will give a superior benefit in palm's growth and disease suppression, application of T4 will also assist planters to minimize GanoCare® application frequency from 12 rounds/year to 4 rounds/year, which will minimize their time, labor, and expenses of fertilizer application, thus maintaining their profitability.

Nutritional status of a plant gives a major impact towards its tolerance to disease. Nutrient status will greatly affect the histological and morphological properties of a plant and, therefore, the responses to pathogen attack. Apart from chemical and biological development of resistant progenies, integrated *Ganoderma* management through manipulation of nutrient uptake is seen as an effective strategy to reduce incidence of *Ganoderma*. Although this approach is yet to be a cure for the BSR disease, it has proven able to arrest the spread of disease in oil palm, hence minimizing the economic loss faced by plantations. To improve the quality of GanoCare®, further testing in the different areas of oil palm plantation shall be carried out, with the strategy to achieve better disease reduction than in present study.

## 4. Conclusion

Increased height, bulb diameter, leaf area, chlorophyll content, photosynthesis rate, and fresh weight and dry weight of leaf, root, and bole of oil palm seedlings have been observed in the nursery trial after application of GanoCare®. Not only as a successful growth enhancer, but the potential use of GanoCare® as an inhibitor of BSR disease has also been established in this study. Pathological analysis of nursery and field trials unveiled the ability of GanoCare® to suppress the BSR incidence, with the control seedlings being the most severely affected throughout the study period. There was prominent alleviation of BSR incidence in seedlings that were treated with GanoCare® compared to the untreated. At the end of experiment, lowest percentage of BSR incidence in the nursery and field was found in seedlings given combination of pretreatment and continuous treatment of 30 g/three months (T4) with incidence of 72.22 and 26.67%, while control seedlings of both trials significantly marked the same maximum percentage of 100%, thus giving significant disease reductions of 77.78 and 82.36%, respectively. Despite the encouraging results obtained from this study, research on the effect of GanoCare® towards the biochemical responses of oil palms should be further undertaken. Future research should clarify whether GanoCare® has the ability to alter the activities of enzymes involved in the metabolic defense. Molecular studies involving gene expression should be undertaken to identify the defense-related mechanism involved.

## Figures and Tables

**Table 1:** GanoCare® treatment in the nursery^a^ and field^b^ trial.

Treatments		^*∗*^Total amount (g/palm)	
		Nursery	Field
CT	Control	0	0
T1	Pretreatment only, with 10 g at monthly interval	30	60
T2	Pretreatment only, with 30 g at three-month interval	30	60
T3	Pretreatment + continuous treatment, with 10 g at monthly interval	110	270
T4	Pretreatment + continuous treatment, with 30 g at three-month intervals	120	270

^a^Treatment used in experiments of vegetative growth, physiological parameters, and suppression of BSR disease in nursery. ^b^Treatment used in pathological study in field trial. ^*∗*^Total amount of GanoCare® received by each seedling throughout the whole period of experiment.

**Table 2:** Effect of GanoCare® on growth of oil palm seedlings^a^.

Trt.	Height (cm)	Bulb diameter (mm)	Leaf area (cm^2^)	Fresh weight (g)			Dry weight (g)		
				Leaf	Bole	Root	Leaf	Bole	Root
CT	61.93^c^	37.84^c^	1491.60^b^	69.07^b^	64.65^d^	107.06^b^	39.07^b^	22.14^c^	40.84^b^
T1	67.92^b^ (10)	39.09^bc^ (3)	1822.49^a^ (22)	80.44^ab^ (17)	76.51^bc^ (18)	121.02^ab^ (13)	44.61^ab^ (14)	28.55^b^ (29)	49.23^ab^ (21)
T2	68.34^b^ (10)	40.76^ab^ (8)	1817.84^a^ (22)	87.83^ab^ (27)	74.86^c^ (16)	118.92^ab^ (11)	40.93^b^ (5)	28.54^b^ (29)	51.49^ab^ (26)
T3	74.81^a^ (21)	41.71^a^ (10)	1886.22^a^ (27)	89.76^a^ (30)	83.27^ab^ (29)	134.66^a^ (26)	48.21^a^ (23)	31.56^ab^ (43)	55.37^a^ (36)
T4	76.48^a^ (23)	41.83^a^ (11)	1916.18^a^ (29)	94.02^a^ (36)	86.69^a^ (34)	135.6^a^ (27)	47.72^a^ (22)	33.02^a^ (49)	55.57^a^ (36)

^a^Values are means of 6 replicates; the same letters within samples in a column for a given parameter are not significantly different according to least significant difference (LSD) at *p* < 0.05. Values in parenthesis are increment percentage relative to the controls.

**Table 3:** Percentage of infected tissues and disease severity of root and bole index in nursery and field trial^a^.

	Root tissue decay (%)		^b^DSIR (%)		Bole tissue decay (%)		^c^DSIB (%)	
	Nursery	Field	Nursery	Field	Nursery	Field	Nursery	Field
CT	99.65^a^	94.55^a^	100.00^a^	95.00^a^	99.44^a^	93.76^a^	100.00^a^	95.00^a^
T1	80.47^ab^	33.49^b^	84.72^ab^	35.00^b^	60.49^b^	29.52^b^	69.44^b^	31.67^b^
T2	75.96^b^	28.74^b^	80.56^abc^	31.67^b^	58.28^b^	24.98^b^	65.28^b^	28.33^b^
T3	71.44^b^	25.19^b^	75.00^bc^	30.00^b^	39.29^bc^	21.74^b^	75.00^bc^	26.67^b^
T4	62.49^b^	18.32^b^	59.72^c^	23.33^b^	28.74^c^	14.63^b^	37.50^c^	18.33^b^

^a^Values are means of 6 replicates in nursery and 5 replicates in field; the same letters within samples in a column for a given parameter are not significantly different according to least significant difference (LSD) at *p* < 0.05. ^b^DSIR = disease severity of root index; ^c^DSIB = disease severity of bole index.

**Table 4:** Disease incidence, area under the disease progress curve, and disease reduction of different treatments in nursery and field trial^a^.

	Disease incidence (%)		^b^AUDPC (unit^2^)		Disease reduction (%)	
	Nursery	Field	Nursery	Field	Nursery	Field
CT	100.00 ± 0.00^a^	100 ± 0.00a	450.00 ± 48.50^a^	800 ± 59.16^a^	—	—
T1	94.44 ± 5.55^ab^	40.00 ± 12.47^b^	294.44 ± 44.24^b^	220 ± 98.23^b^	32.50	69.18
T2	94.44 ± 5.55^ab^	40.00 ± 19.44^b^	216.67 ± 48.49^bc^	220 ± 107.94^b^	49.58	69.82
T3	83.33 ± 11.39^ab^	33.33 ± 10.54^b^	161.11 ± 45.88^cd^	150 ± 72.46^b^	63.47	78.93
T4	72.22 ± 10.24^b^	26.67 ± 12.47^b^	105.56 ± 31.53^d^	120 ± 75.17^b^	77.78	82.36

^a^Values are means of 6 replicates in nursery and 5 replicates in field ± standard error; the same letters within samples in a column for a given parameter are not significantly different according to least significant difference (LSD) at *p* < 0.05. ^b^AUDPC = area under the disease progress curve.

## Data Availability

The data used to support the findings of this study are provided in the figures and tables.
